# Orodispersible Hydrogel Film Technology for Optimized Galantamine Delivery in the Treatment of Alzheimer’s Disease

**DOI:** 10.3390/gels11080629

**Published:** 2025-08-10

**Authors:** Dilyana Georgieva, Ivana Bogdanova, Rositsa Mihaylova, Mariela Alexandrova, Silvia Bozhilova, Darinka Christova, Bistra Kostova

**Affiliations:** 1 Department of Pharmaceutical Technology and Biopharmacy, Faculty of Pharmacy, Medical University of Sofia, 1000 Sofia, Bulgaria; bogdanovav.iv@gmail.com; 2 Department of Pharmacology, Pharmacotherapy and Toxicology, Faculty of Pharmacy, Medical University of Sofia, 1000 Sofia, Bulgaria; rositsa.a.mihaylova@gmail.com; 3 Institute of Polymers, Bulgarian Academy of Sciences, 1113 Sofia, Bulgaria; m.alexandrova@polymer.bas.bg (M.A.); s.bozhilova@polymer.bas.bg (S.B.); dchristo@polymer.bas.bg (D.C.)

**Keywords:** galantamine hydrobromide, Alzheimer’s disease therapy, orodispersible films, polyoxazolines, drug delivery

## Abstract

Alzheimer’s disease is the most widespread neurodegenerative disease in the world. Galantamine hydrobromide (GH) is one of the drugs used to treat mild to moderate dementia of the Alzheimer type. Due to the fact that the specificity of the disease requires maximally facilitated intake, orodispersible films present such an opportunity. In the present study orodispersible films based on poly(2-ethyl-2-oxazoline) as well as partially hydrolyzed poly(2-ethyl-2-oxazoline) were prepared and studied as delivery systems for GH. Two samples of partially hydrolyzed PEtOx were synthesized—one of relatively low degree of hydrolysis and another one of relatively high degree of hydrolysis, and studied by Nuclear Magnetic Resonance (NMR). Cytotoxicity assay was performed that validated the low hydrolyzed derivative as biocompatible polymer that maintained desirable physicochemical characteristics without compromising the safety, thereby it was selected for further research. The films were prepared by the solution casting method and characterized by different methods. FTIR was used to determine the potential interactions between the galantamine molecule and the film components. Based on the Thermogravimetric Analysis (TGA) conducted, it was concluded that all films were sufficiently thermally stable, as the component decomposition stage (after initial solvent removal) began above 180 °C. The polymer films were further characterized with the determination of Shore hardness and the results showed that the films containing glycerol as a plasticizer exhibited higher hardness compared to those with PEG as a plasticizer. The disintegration time of the films was determined visually using Petri dishes and it was found that the films disintegrated within the range of 0.52 to 1.58 min, fully meeting the pharmacopoeial requirements. GH release profiles in PBS at 37 °C were obtained, and it was found that by the second minute, 80–90% of the drug were released from the different films, and the release followed an anomalous diffusion mechanism (Case II).

## 1. Introduction

Many modern therapies are designed as a “one-size-fits-all” treatment that may be successful for some patients but completely ineffective for others. Precision medicine is an innovative approach based on the concept of providing health care tailored to the patient profile and disease characteristics [1]. The main goal is optimization of the diagnosis, prevention and treatment of different conditions [2,3,4,5]. Geriatric patients are one of the groups most in need of personalized therapy. Dysphagia is very typical for these patients, and multimorbidity, polypharmacy and poor compliance further complicate the therapy. In addition, fluctuations in the drugs’ pharmacokinetics are observed [6,7,8].

Orodispersible films (ODFs) are an attractive new dosage form that is suitable for the above-mentioned target group. They are subjected to extensive studies and are rapidly entering practice [6,7,8]. ODFs are monolayer or multi-layer, small-sized thin strips prepared from suitable polymers. They are usually formulated to disintegrate almost instantaneously prior contact with the oral mucosa and rapidly release the incorporated drug in the mouth, forming a fine suspension or solution in saliva without chewing/water intake [9]. The oral mucosa is supplied with blood, which allows drugs to be absorbed directly and enter the systemic circulation, thus avoiding the “first-pass” effect, which is a prerequisite for improved bioavailability. It is important to keep in mind that, depending on the technological scheme used, both a systemic and a local effect can be achieved by applying ODFs [10,11,12]. The film-forming polymer is the most important component of ODFs. It is important to select an optimal composition in order to achieve a balance between the disintegration time and the mechanical properties of ODFs [13,14,15,16].

Polyoxazolines (POx) are innovative biomaterials that show similar and even better properties compared to well-known analogues, especially PEG. They are biocompatible and can be used for a number of biomedical applications [17,18,19,20]. The use of polyoxazolines for drug modification and delivery has attracted increasing research interest in recent years due to their favorable properties. One aspect of these studies relates to the incorporation of drugs into the polymer chain with the aim of releasing them through hydrolytic degradation [19]. Poly(2-oxazolines) can be synthesized in a controlled manner using the cationic ring-opening polymerization (CROP) method [18]. In recent years, research has been carried out related to the partial hydrolysis of poly(2-ethyl-2-oxazoline) (PEtOx), which leads to the preparation of poly[(2-ethyl-2-oxazoline)-*co*-poly(ethyleneimine)] (PEtOx-*co*-PEI) copolymers. They combine the properties of both POx and polyethyleneimine. These copolymers respond to external stimuli such as temperature and pH and can be used as drug delivery systems [21,22,23,24]. As a result of the partial hydrolysis, secondary amino groups are introduced in the structure of the partially hydrolyzed poly(2-ethyl-2-oxazoline) (PEtOx-h), which enables subsequent structural modifications, expanding the synthetic flexibility [25]. In addition, factors influencing bio tolerability and especially cytotoxicity of partially hydrolyzed PEtOx are investigated in view of the potential biomedical application [26,27].

Alzheimer’s disease is the most widespread neurodegenerative disease in the world. It is characterized by changes in the brain that lead to the deposition of certain proteins and is the most common cause of dementia—a gradual deterioration of memory, thinking, behavior and social skills. Although currently available drugs do not prevent or cure the disease, they delay cognitive decline, institutionalization, thus improving the quality of life and providing economic benefits.

Galantamine hydrobromide (GH) is one of the drugs used to treat mild to moderate dementia of the Alzheimer type. Galantamine is supplied orally as a solution, tablets and capsules. As the specificity of the disease requires maximally facilitated intake, ODFs are a dosage form gaining more and more popularity in recent years due to the fact that they possess the appropriate characteristics to increase the effectiveness and personalize the therapy. Poly(2-ethyl-2-oxazoline), as well as the partially hydrolyzed poly(2-ethyl-2-oxazoline), are innovative polymers with unique properties superior to conventionally used polymers. They are suitable as potential drug delivery systems. Poly(2-ethyl-2-oxazoline) has been approved by the FDA for use as adhesive, and a side-chain rotigotine-loaded PEtOx has gained approval to start Phase 1 clinical trials for the treatment of Parkinson’s disease [28,29]. No information has been found in the literature to date on the preparation of orodispersible films for the delivery of galantamine based on poly(2-ethyl-2-oxazoline) as well as partially hydrolyzed poly(2-ethyl-2-oxazoline), also there are currently no ODFs with GH on the market, which was the major reason for conducting the present study.

## 2. Results and Discussion

### 2.1. Preparation of Partially Hydrolyzed Poly(2-ethyl-2-oxazoline)

**Figure 1:**
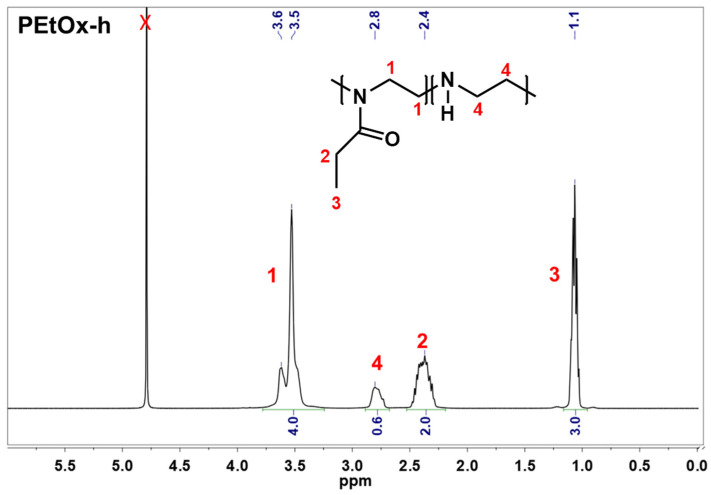

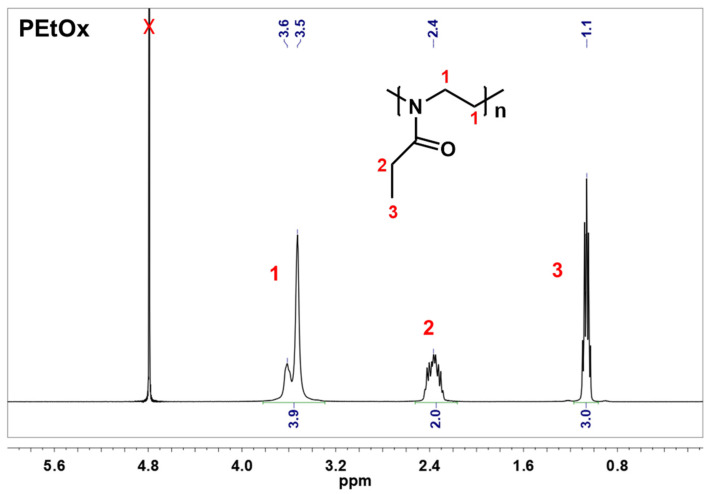
^1^H NMR spectra (solvent D_2_O) of PEtOx and PEtOx-h.

Following the steps of the methodology detailed in section Methods, two samples of partially hydrolyzed PEtOx were synthesized to be used in the present investigation—one of relatively low degree of hydrolysis and another one of relatively high degree of hydrolysis. Nuclear magnetic resonance (NMR) was used to characterize and calculate the degree of hydrolysis of the obtained partially hydrolyzed poly(2-ethyl-2-oxazoline). In Figure 1 are compared the proton spectra of PEtOx and PEtOx-h. In the PEtOx spectrum, the signals for the methyl and methylene protons from the ethyl group at 1.06 ppm and 2.37 ppm, respectively, and the methylene groups from the main chain at 3.53–3.62 ppm were clearly distinguished. After hydrolysis, a signal at 2.80 ppm, characteristic of the methylene protons of the polyethyleneimine units, appeared in the spectrum of the obtained PEtOx-h. From the ratio of the integrated areas of the signals assigned the methylene groups at peaks 1 and 4, the degree of hydrolysis of 13.6 and 60.7% was estimated for the low and high hydrolyzed sample, respectively. The samples were labeled PEtOx-h14 and PEtOx-h61, with the number after the code indicating the degree of hydrolysis of the respective copolymer.

### 2.2. Citotoxicity Assay

**Figure 2:**
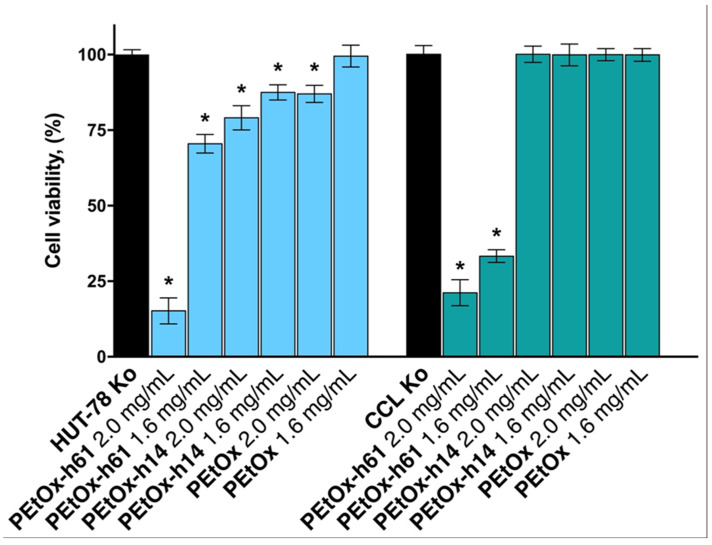
Effects of the tested non-hydrolyzed PEtOx and partially hydrolyzed PEtOx-h14 and PEtOx-h61 polymers on cell viability against normal murine fibroblast cells (CCL-1) and cutaneous T-cell lymphoma in vitro model (HUT-78) after continuous 72 h exposure to two different concentrations. All experiments were run in triplicate and data were expressed as mean ± SD. Statistical significance of the data was assessed via a one-way ANOVA (* *p* ≤ 0.01 vs. untreated control).

The results obtained from the cytotoxicity assay are presented in Figure 2. Based on the MTT assay data assessing cell viability in HUT-78 and CCL-1 cell lines, a comparative analysis of the effects of the non-hydrolyzed polymer PEtOx and two partially hydrolyzed derivatives, PEtOx-h14 and PEtOx-h61 revealed a clear correlation between the degree of hydrolysis and cytotoxicity. This analysis informed the decision to select PEtOx-h14 variant for orodispersible hydrogel film formulation, primarily due to its superior biocompatibility profile.

In the T-cell lymphoma-derived HUT-78 line, the non-hydrolyzed PEtOx displayed excellent cytocompatibility, with cell viability remaining at 99.5% at 1.6 mg/mL and slightly lower (87%) at 2.0 mg/mL. This demonstrated that PEtOx, in its native form, was minimally cytotoxic across both concentrations tested.

PEtOx-h14 also showed high cell viability—87.5% at 1.6 mg/mL and 79.1% at 2.0 mg/mL—indicating that minimal hydrolysis did not significantly compromise cell viability, and its toxicity remained close to that of the unmodified PEtOx. In contrast, PEtOx-h61 exhibited pronounced cytotoxicity, reducing cell viability to 70.5% at 1.6 mg/mL and further down to 15.2% at 2.0 mg/mL. This steep decline suggests that higher hydrolysis leads to increased generation of functional groups—likely carboxylic acids or related moieties—that may interfere with cellular homeostasis or induce membrane disruption.

The pattern observed in HUT-78 was reinforced in the murine fibroblast CCL-1 line. PEtOx maintained nearly complete viability at both concentrations (100% at 2.0 mg/mL and 99.9% at 1.6 mg/mL), further confirming its non-toxicity. PEtOx-h14 showed similarly exceptional biocompatibility, with viability rates of 100% (1.6 mg/mL) and 99.9% (2.0 mg/mL). In contrast, PEtOx-h61 again demonstrated significant cytotoxicity, reducing cell viability to 33.3% at 1.6 mg/mL and as low as 21.2% at 2.0 mg/mL.

The data consistently show a toxicity gradient that correlates with the degree of hydrolysis: PEtOx ≈ PEtOx-h14 >> PEtOx-h61. This trend was particularly stark in the CCL-1 line, where PEtOx-h61 exhibited the highest toxicity, indicating that hydrolysis-induced chemical modifications markedly influence cell compatibility.

Given these findings, PEtOx-h14 was selected for incorporation into orodispersible hydrogel film formulations. It offers a balance between retaining some hydrolytic modification—potentially useful for improving solubility or mucoadhesion—while minimizing adverse effects on cellular health. Its low cytotoxicity across two distinct cell lines supports its suitability for further pharmaceutical development, particularly in dosage forms aimed at vulnerable populations such as Alzheimer’s patients.

In conclusion, the MTT data validated PEtOx together with its low hydrolyzed derivative PEtOx-h14 as biocompatible polymers that maintain desirable physicochemical characteristics without compromising safety, thereby justifying their selection for clinical formulation use.

### 2.3. Preparation of Galantamine-Loaded and Not-Loaded Orodispersible Films

The initial composition of the solutions used for preparation of the different ODFs is presented in Table 1.

In our preliminary studies, we have investigated the influence of the plasticizer and the film-forming polymer on the quality and disintegration of ODFs. The quality, thickness, weight, folding resistance and disintegration time of the different compositions were determined. The conducted studies proved that the plasticizer and the film-forming polymer have a significant influence on the studied parameters. We used the obtained information in the present study to obtain films with optimal characteristics to be used for the delivery of galantamine hydrobromide.

**Figure 3:**
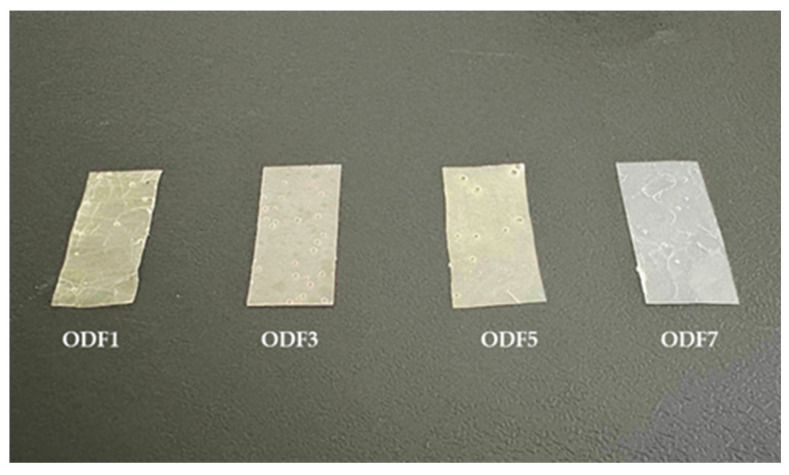
Images of the prepared ODFs with incorporated GH.

The preparation of ODFs requires the use of water-soluble polymers in order to achieve fast disintegration in the saliva. The polymers used in this study were PEtOx and PEtOx-h14, and glycerol and PEG 400 were used as plasticizers. The function of maltodextrin (MD) was to improve the flexibility of the prepared films [30]. The visual inspection confirmed the good quality of the obtained films, which were elastic (Figure 3).

### 2.4. Characterization of the Obtained Orodispersible Films

#### 2.4.1. Fourier Transform Infrared Spectroscopy (FTIR)

**Figure 4:**
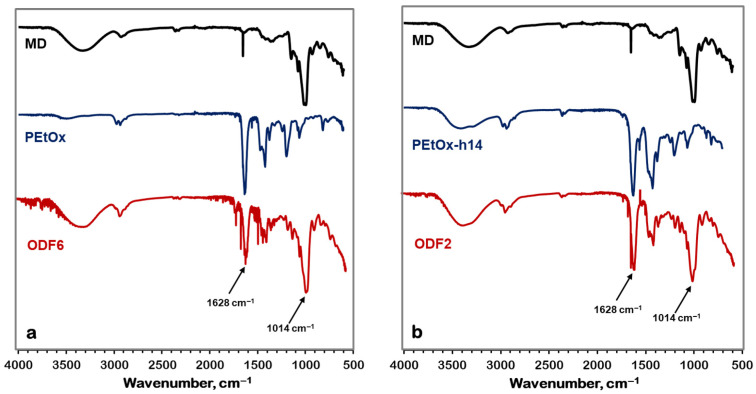
(**a**) FTIR spectra of MD, PEtOx and ODF6 without incorporated galantamine; (**b**) FTIR spectra of MD, PEtOx-h14 and ODF2 without incorporated galantamine.

FTIR was used to determine the potential interactions between the galantamine molecule and the film components. Figure 4 presents the spectra of PEtOx, maltodextrin, and a film without incorporated galantamine.

A distinct peak at 1628 cm^−1^ was observed for the >C=O stretching vibrations of the amide group of PEtOx is observed in the PEtOx spectrum. The signals appearing at 1420 cm^−^^1^ and 2940/2976 cm^−1^ correspond to the stretching and bending vibrations of >C-H respectively. The spectrum of maltodextrin shows characteristic peaks at 3350 cm^−1^ (OH), 2928 cm^−1^ (C-H stretching), 1014 cm^−1^ (C-O stretching), 1338 cm^−1^ (OH bending), and 848 cm^−1^ (OH stretching). From the FTIR spectrum presented in Figure 4, it is clear that in the obtained film, the main peaks of PEtOx and maltodextrin were observed at the same wavelengths as in the spectra of the individual components. The results in Figure 4 show that the spectrum of PEtOx-h14 comprises a peak at 3277 cm^−1^, characteristic of N-H groups, in addition to the bands of the main polymer. In the film, the peak for C-O-C vibrations, characteristic of maltodextrin, shifts from 1014 cm^−1^ to 1022 cm^−1^, suggesting interaction of maltodextrin with the hydrolyzed part of the PEtOx.

**Figure 5:**
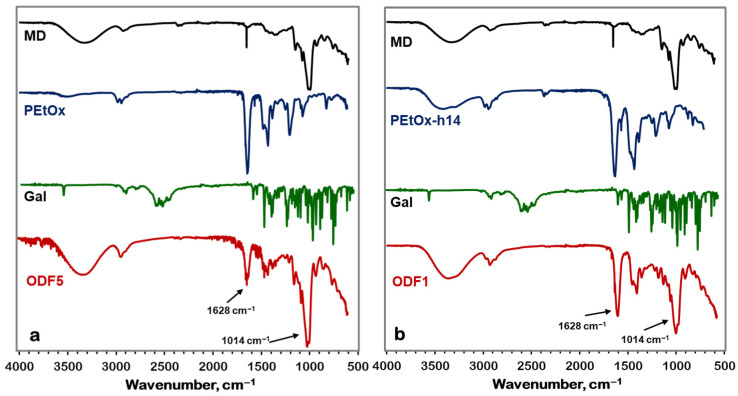
(**a**) FTIR spectra of MD, PEtOx and ODF5 with incorporated galantamine; (**b**) FTIR spectra of MD, PEtOx-h14 and ODF1 with incorporated galantamine.

Figure 5 presents the spectrum of galantamine, which is very characteristic. The most specific peaks are at 1016 cm^−1^, 2834 cm^−1^, 3429 cm^−1^, and 3559 cm^−1^, corresponding to the stretching vibrations of C-O-C, >C-H, >N-H, and –O-H bonds, respectively. In the obtained films, alongside the intense peaks of the two main components, the peak characteristic of galantamine at 1016 cm^−1^ is clearly visible. From the spectrum, it is evident that the C-O-C vibration peak shifts to 1022 cm^−1^. This confirms that the drug molecule interacts with the hydrolyzed part of PEtOx, similar to maltodextrin.

#### 2.4.2. Thermogravimetric Analysis (TGA)

TGA was conducted for the films that used glycerin as a plasticizer in order to study the compatibility between the individual components in the film. These films were selected due to the fact that glycerin is widely used in the pharmaceutical practice as a multifunctional excipient. It is preferred as a plasticizer in the preparation of films because it provides greater flexibility, good dispersion in the oral cavity and a pleasant taste.

**Figure 6:**
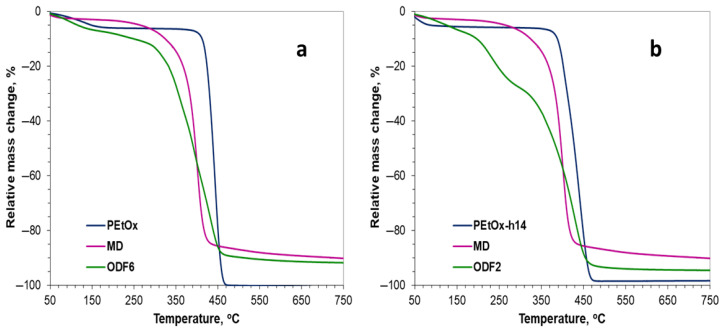
(**a**) TGA curves of MD, PEtOx and ODF6 without incorporated galantamine; (**b**) TGA curves of MD, PEtOx-h14 and ODF2 without incorporated galantamine.

Figure 6a presents the characteristic thermograms of pure poly(2-ethyl-2-oxazoline), maltodextrin, and the prepared film. The observed weight loss in the range of 50–150 °C was due to the release of the residual solvent, in this case, water. In the PEtOx thermogram, the main weight loss (94%) occurred between 400–470 °C, leaving no non-combustible residue. Unlike PEtOx, maltodextrin degraded over a much wider temperature range of 250–500 °C (82% weight loss). At the end of the analysis, about 10% non-combustible residue remained. Each step in the thermogram corresponds to the products present in the sample. Several stages of decomposition were observed, with the two main ones being 250–350 °C, corresponding to maltodextrin (30% weight loss), and 350–500 °C, corresponding to PEtOx (53% weight loss). The non-combustible residue was about 10%.

From the thermogram presented in Figure 6b, it is evident that the partially hydrolyzed PEtOx degraded over a slightly wider temperature range compared to PEtOx, namely 380–480 °C (93% weight loss). No non-combustible residue was observed; complete destruction occurred. In the film’s thermogram, again, several decomposition stages were observed, with the main ones being 250–300 °C, corresponding to maltodextrin (20% weight loss), and 300–500 °C, corresponding to PEtOx-h14 (65% weight loss). The non-combustible residue amounted to 5%. The much wider decomposition interval of the PEtOx part was due to the interaction between N-H in the hydrolyzed part of polyoxazoline and the other components in the film.

**Figure 7:**
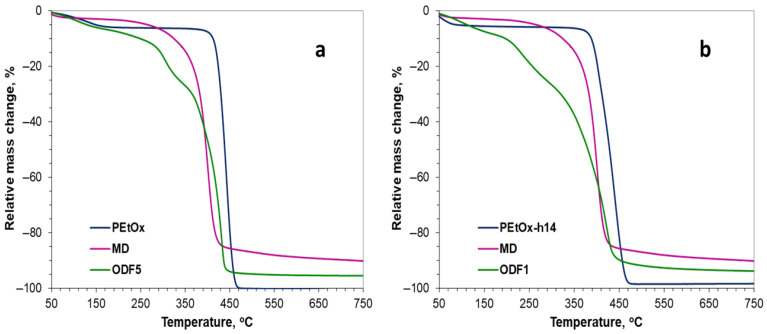
(**a**) TGA curves of MD, PEtOx and ODF5 with incorporated galantamine; (**b**) TGA curves of MD, PEtOx-h14 and ODF1 with incorporated galantamine.

In the thermogram presented in Figure 7a, several stages of degradation were again observed, which corresponded to the components in the film. The main ones were 180–350 °C, corresponding to maltodextrin (19% mass loss) and 350–480 °C, corresponding to PEtOx (69% mass loss). The non-combustible residue was about 5%. The significantly higher rate of degradation of the film was obviously due to the interaction of the drug with the other components of the film.

The thermogram of the film based on partially hydrolyzed poly(2-ethyl-2-oxazoline) presented in Figure 7b shows several decomposition stages. The main ones were 180–300 °C, corresponding to maltodextrin (18% weight loss), and 300–500 °C, corresponding to PEtOx-h14 (65% weight loss). The non-combustible residue was about 5%. The curve is multi-stage and complex, due to the interaction of the hydrolyzed polyoxazoline parts with maltodextrin and, most notably, galantamine.

Based on the analysis, it can be concluded that all films were sufficiently thermally stable, as the component decomposition stage (after initial solvent removal) began above 180 °C.

#### 2.4.3. Shore Hardness Assessment

The polymer films were further characterized with the determination of Shore hardness. It is known that higher values on the Shore scale indicate greater resistance and, therefore, harder materials. All the tested films demonstrated high values, indicative of their good mechanical durability. Based on the results presented in Table 2, it can be concluded that the films containing glycerol as a plasticizer exhibited higher hardness compared to those with PEG as a plasticizer.

#### 2.4.4. Disintegration

The disintegration time of the films was determined visually using Petri dishes, and the average values obtained from three experiments for each film are presented in Table 3. Currently, there are no official guidelines for the disintegration time of rapidly disintegrating oral films. Therefore, the specifications for orodispersible tablets included in the European Pharmacopoeia were used, which require disintegration within 3 min.

The presented data indicate that the films disintegrated within the range of 0.52 to 1.58 min, fully meeting the pharmacopoeial requirements. Typically, the disintegration time is a function of the film’s composition and varies depending on the formulation.

It can be observed that the films based on partially hydrolized polyoxazolines disintegrated faster compared to the films based on pristine polyoxazoline. Furthermore, the addition of glycerol as a plasticizer increased the disintegration time, which was also observed by other scientific groups [31,32].

#### 2.4.5. In Vitro Drug Release Study

**Figure 8:**
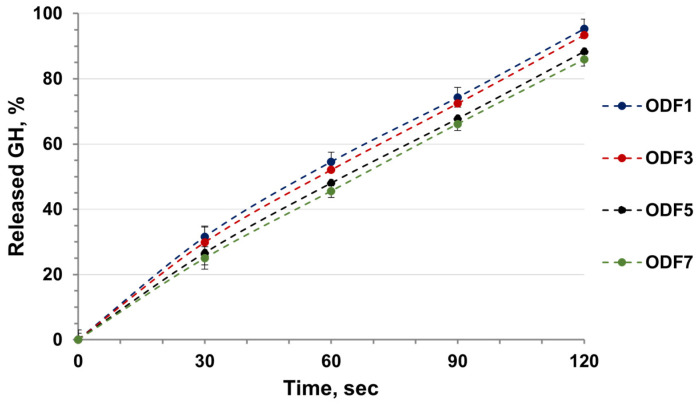
In vitro galantamine release study of the prepared films at pH 6.8.

The results obtained from the in vitro drug release study are presented in Figure 8.

From the results it is obvious that by the second minute, 80–90% of the drug were released from the different films. Comparing the individual compositions showed that the fastest release of the incorporated galantamine was observed from the films based on partially hydrolyzed poly(2-ethyl-2-oxazoline). These results are expected, given the disintegration times of the different films.

Based on the conducted studies, it can be concluded that the prepared films based on polyoxazolines have good characteristics and hold potential as drug delivery systems in cases where a rapid effect is desired.

#### 2.4.6. Drug Release Kinetics

To study the drug release profiles from the prepared films, various kinetic models were applied. The correlation coefficient (R^2^) was used to compare the different models, where a value closer to 1 indicated a better correlation. Table 4 presents the correlation coefficients (R^2^), constants of the different models, and the release exponent (*n*) obtained after fitting the experimental data for galantamine release from the tested films.

It was established that the release of galantamine from the samples was best described by zero-order kinetics (R^2^ > 0.99). These results indicate that drug release from the films was a function of time only and occurred at a constant rate, regardless of the concentration of the active agent.

The Korsmeyer-Peppas model was also used to establish the drug release from the polymer systems. The release exponent (*n*) in this model indicates the nature of the drug release mechanism. In occasions when *n* ≤ 0.5, the release is dominated by Fickian diffusion; when 0.5 ≤ *n* ≤ 1, the release follows an anomalous diffusion mechanism (non-Fickian diffusion—Case II); and when *n* > 1, the release is based on a complex transport mechanism (super-case-II transport) [33]. From the n values presented in Table 4, it can be concluded that the release followed an anomalous diffusion mechanism (Case II), the drug release rate corresponded to zero-order kinetics (*n* ~ 1), and the driving force of the process was the swelling or the relaxation of the polymer chains. Similar results obtained for the drug release kinetics are noted and discussed by other scientific groups studying ODFs [34,35,36].

## 3. Conclusions

In the present work orodispersible films based on poly(2-ethyl-2-oxazoline) as well as partially hydrolyzed poly(2-ethyl-2-oxazoline) were prepared and studied as delivery systems for GH. NMR was used to characterize and calculate the degree of hydrolysis of the obtained partially hydrolyzed poly(2-ethyl-2-oxazoline). The performed cytotoxicity assay validated PEtOx and PE-tOx-h14 as biocompatible polymers that maintained desirable physicochemical characteristics without compromising the safety, thereby they were used for further studies. The films were prepared by the solution casting method using glycerol and PEG 400 as plasticizers, and were characterized by different methods. FTIR was used to determine the potential interactions between the galantamine molecule and the film components and based on the TGA conducted, it was concluded that all films were sufficiently thermally stable. The films disintegrated within the range of 0.52 to 1.58 min, fully meeting the pharmacopoeial requirements. GH release profiles in PBS at 37 °C were obtained, and it was found that by the second minute, 80–90% of the drug were released from the different films, and the re-lease followed an anomalous diffusion mechanism (Case II). The results obtained indicate that the prepared films based on PEtOx have a potential as drug delivery systems for GH in the treatment of Alzheimer’s disease.

## 4. Materials and Methods

### 4.1. Materials

GH was supplied by Sopharma AD (Sofia, Bulgaria). Maltodextrin, glycerol, and PEG 400 (pharmaceutical grade, MW ~ 400 Da) were obtained from SoleChem (Milano, Italy). Poly(2-ethyl-2-oxazoline) (PEtOx) of reported average molecular weight ~500,000 g/mol and dispercity Ð = 3–4 was purchased from Sigma Aldrich (St. Louis, MO, USA).

### 4.2. Methods

#### 4.2.1. Synthesis and Characterization of Partially Hydrolyzed Poly(2-ethyl-2-oxazoline)

- Synthesis of partially hydrolyzed poly(2-ethyl-2-oxazoline)

Partially hydrolyzed poly(2-ethyl-2-oxazoline) (PEtOx-h) samples investigated in this work were prepared by acidic hydrolysis of PEtOx in a procedure adapted from the literature [25,37]. In brief, 30 g of PEtOx were dissolved in 200 mL of deionized water using a magnetic stirrer. The solution was transferred in a round-bottomed flask equipped with condenser and dropping funnel. Hydrolysis was carried out by adding dropwise the needed amount of 10% hydrochloric acid to the polymer solution and stirring the reaction mixture on a magnetic stirrer for 1.5 h at 100 °C (reflux). Following the same procedure, two samples of target degree of hydrolysis 15 and 60% mol were synthesized using corresponding equimolar amount of HCl. After hydrolysis, cooled reaction mixture was neutralized with aqueous solution of sodium hydroxide (5 N) and dialyzed against deionized water using dialysis membrane of MWCO 12,000 Da. Finally, purified PEtOx-h was isolated from the solution by lyophilization. Two samples of partially hydrolyzed PEtOx were synthesized and labeled PEtOx-h14 and PEtOx-h61, with the number after the code indicating the approximate degree of hydrolysis of the respective sample.

- Nuclear magnetic resonance (NMR)

NMR was used to follow the extend of hydrolysis of poly(2-ethyl-2-oxazoline) and to determine the composition of the obtained partially hydrolyzed copolymers. The study was performed on a Bruker Avance III operating at 400 MHz spectrometer (Bruker Corporation, Billerica, MA, USA) using deuterated water (D_2_O) as a solvent. The chemical shifts δ are reported in parts per million (ppm), relative to the residual nondeuterated solvent peak.

- Citotoxicity assay

*Cell lines and culture conditions*

The in vitro cytotoxicity and biocompatibility of the tested materials were evaluated using two distinct cell lines: normal murine fibroblasts (CCL-1) and malignant human cutaneous T-cell lymphoma cells (HUT-78). Both lines were obtained from the German Collection of Microorganisms and Cell Cultures (DSMZ GmbH, Braunschweig, Germany). CCL-1 cells were cultured in EMEM supplemented with 10% horse serum, while HUT-78 cells were maintained in IMDM enriched with 20% fetal bovine serum. All cultures were grown under standard incubation conditions of 37 °C in a humidified atmosphere containing 5% CO_2_.

In vitro *MTT colorimetric assay*

The growth-inhibitory potential of the polymeric material was assessed using the established Mosmann MTT assay, a standard method for evaluating cell viability. Cells in the exponential growth phase were collected and seeded into 96-well plates (100 μL per well) at densities of 3 × 10^5^ for HUT-78 (suspension culture) and 1.5 × 10^5^ for CCL-1 (adherent cells). After a 24-h incubation period, cultures were treated with two concentrations of the polymer—filtered and unfiltered—corresponding to the final well concentrations of 2 mg/mL and 1.6 mg/mL.

Following 72 h of exposure, filter-sterilized MTT solution (5 mg/mL in PBS) was introduced to each well. The incubation was continued for an additional 2 to 4 h to allow for the formation of insoluble purple formazan crystals. These were subsequently solubilized in an isopropanol solution containing 5% formic acid. The absorbance was then measured at 550 nm using a Labexim LMR-1 microplate reader. The readings were corrected for background using wells containing only MTT and isopropanol, and the results were expressed relative to the untreated control, set as 100% cell viability.

#### 4.2.2. Preparation of Galantamine-Loaded and Non-Loaded Orodispersible Films

2 g of PEtOx or PEtOx-h14 were dissolved in 40 mL of water using a magnetic stirrer. A solution of maltodextrin was prepared by mixing 1 g with 40 mL of water. The two solutions were then mixed and homogenized. The resulting solution was divided into four equal parts in beakers. Plasticizer glycerin (0.135 g) was added to two of the solutions, and PEG 400 (0.225 g) to the other two samples. Galantamine (0.03 g) was added to one of the solutions with plasticizer glycerin and to one of the solutions with PEG 400. The obtained solutions were used for the preparation of the films by the solution casting method. The films were initially dried at room temperature followed by drying in vacuum until constant weight using CaCl_2_ as a drying agent.

#### 4.2.3. Characterization of the Obtained Orodispersible Films

- Fourier transform infrared spectrometry (FTIR)

Fourier transform infrared spectra were recorded using an IRAffinity-1 spectrophotometer (Shimadzu Company, Kyoto, Japan) equipped with a MIRacleTM ATR accessory (diamond crystal; PIKE Technologies, Madison, WI) providing infrared penetration depth into the sample of about 2 μm. The film samples were analyzed in the wavelength range from 4000 to 700 cm^−1^, performing 50 scans at a resolution of 4 cm^−1^. FTIR measurements were repeated on 3 different spots of the same film.

- Thermogravimetric analysis (TGA)

The bulk thermal stability and compositional properties of the films were evaluated using TGA. The thermal properties of the films were investigated using a TGA-4000 Perkin Elmer apparatus (PerkinElmer, Inc., Waltham, MA, USA). Film samples weighing 12 to 14 mg were tested from 40 to 650 °C under nitrogen flow at a heating rate of 10 °C/min.

- Shore (A) hardness assessment

The hardness of the films was measured using a Shore Durometer Test Stand, Model TI-AC, SN:4A20J07668 (SAUTER GmbH) with a scale of 0 to 100, with higher values indicating harder material. Samples of orodispersible films of thickness 0.4 ± 0.02 mm were investigated and the mean values of Shore hardness A were obtained from measuring 10 times at different sites.

- Disintegration

The determination was carried out in two media—at pH 1.2 and at pH 6.8. Parts of the films with sizes 1.1 cm/2.5 cm and thickness 0.4 ± 0.02 mm were placed in petri dishes and 2 mL of diluted hydrochloric acid pH 1.2/phosphate buffer pH 6.8 were added. The time when the film disintegrated into fragments was detected.

- In vitro drug release study

The study was performed by immersing the samples in 100 mL of phosphate buffer (pH 6.8). The release was carried out in a thermostated (37° ± 0.5 °C) shaking water bath. At certain time intervals, a 2 mL sample was taken for analysis. After each sampling, the volume was restored with an equivalent amount of phosphate buffer. The amount of galantamine released was determined by UV spectroscopy (Hewlett-Packard 8452 A Diode Array spectrophotometer, Palo Alto, CA, USA).

- Drug release kinetics

The drug release kinetics from the prepared orodispersible polymer films were determined by fitting the experimental data of the in vitro release of galantamine using different kinetic models, presented in Table 5:

Where, M(t) represents the amount of galantamine released at time t; M(∞) is the total amount of galantamine incorporated in the films; k_0_, k_1_, k_H_ and k_KP_ are the zero-order, first-order, Higuchi and Korsmeyer-Peppas constants, respectively; *n* is the release exponent.

## Figures and Tables

**Table 1:** Composition of the prepared ODFs casting solutions.

Code	Component						
	PEtOx-h14 (%)	PEtOx (%)	Maltodextrin (%)	Glycerol (%)	PEG 400 (%)	GH (%)	Water (%)
ODF1	3.3	0	2.7	0.9	0	0.2	92.9
ODF 2	3.3	0	2.7	0.9	0	0	93.1
ODF 3	3.3	0	2.7	0	1.5	0.2	92.3
ODF 4	3.3	0	2.7	0	1.5	0	92.5
ODF 5	0	3.3	2.7	0.9	0	0.2	92.9
ODF 6	0	3.3	2.7	0.9	0	0	93.1
ODF 7	0	3.3	2.7	0	1.5	0.2	92.3
ODF 8	0	3.3	2.7	0	1.5	0	92.5

**Table 2:** Shore (A) hardness values of the studied films.

Sample	Shore (A) Hardness
ODF1	92 ± 2
ODF2	88 ± 2
ODF3	80 ± 3
ODF4	83 ± 2
ODF5	90 ± 4
ODF6	86 ± 2
ODF7	85 ± 3
ODF8	85 ± 3

**Table 3:** Disintegration time of the different films.

Sample	Disintegration Time at pH 1.2 (min)	Disintegration Time at pH 6.8 (min)
ODF1	0.57 ± 0.05	0.58 ± 0.16
ODF3	0.52 ± 0.15	0.55 ± 0.29
ODF5	1.40 ± 0.39	1.58 ± 0.67
ODF7	1.36 ± 0.25	1.52 ± 0.48

**Table 4:** Release kinetics of the studied films.

Sample	Zero Order	First Order	Higuchi	Korsmeyer-Peppas
ODF1	k_0_ = 0.0778 R^2^ = 0.9907	k_1_ = 0.0179 R^2^ = 0.8985	k_H_ = 8.4879 R^2^ = 0.9613	*n* = 0.9593 R^2^ = 0.9977
ODF3	k_0_ = 0.0765 R^2^ = 0.9935	k_1_ = 0.0179 R^2^ = 0.911	k_H_ = 8.3062 R^2^ = 0.9549	*n* = 0.9531 R^2^ = 0.9985
ODF5	k_0_ = 0.0545 R^2^ = 0.9964	k_1_ = 0.0157 R^2^ = 0.9622	k_H_ = 7.838 R^2^ = 0.946	*n* = 0.9386 R^2^ = 0.9995
ODF7	k_0_ = 0.0532 R^2^ = 0.9979	k_1_ = 0.0157 R^2^ = 0.97	k_H_ = 7.6282 R^2^ = 0.9395	*n* = 0.9313 R^2^ = 0.9998

**Table 5:** Different kinetic models.

Kinetic Models	Equation
Zero order	$\frac{M(t)}{M(\infty )}=k_{0}t$
First order	$\frac{M(t)}{M(\infty )}=e^{-k_{1}t}$
Higuchi Diffusion Kinetic Model	$\frac{M(t)}{M(\infty )}=k_{H}t^{\frac{1}{2}}$
Korsmeyer-Peppas model	$\frac{M(t)}{M(\infty )}=k_{KP}t^{n}$

## Data Availability

The original contributions presented in this study are included in the article. Further inquiries can be directed to the corresponding authors.
